# Self-Reported Outcome of Living Kidney Donation Correlates With Perioperative Complications not With Surgical Techniques

**DOI:** 10.1016/j.ekir.2025.06.052

**Published:** 2025-07-02

**Authors:** Martina Koch, Jeannine Wegner, Eike Bormann, Sylvia Kröncke, Sarah Riepenhausen, Philipp Neuhaus, Julian Varghese, Joachim Gerß, Claudia Sommerer, Barbara Suwelack, SOLKID-GNR Registry Group, Anja Mühlfeld, Florian Sommer, Aydin Er, Klemens Budde, Lutz Liefeld, Fabian Halleck, Mira Choi, Julian Stumpf, Juliane Putz, Johannes Stegbauer, Susanne Mende, Mario Schiffer, Katharina Heller, Andreas Kribben, Bernd Jänigen, Johanna Schneider, Rolf Weimer, Hristos Karakizlis, Ulrich Pein, Lutz Fischer, Malte Kluger, Peter Weithofer, Volker Kliem, Martin Zeier, Mandy Schlosser, Gunter Wolf, Thomas Rath, Christian Mönch, Kevin Schulte, Friedrich A. von Samson-Himmelstjerna, Dirk Stippel, Christine Kurschat, Ana Harth, Christian Jungck, Anette Bachmann, Antje Weimann, Martin Nitschke, Figen Cakiroglu, Julia Weinmann-Menke, Birgit Kortus-Götze, Joachim Hoyer, Stephan Kemmner, Manfred Stangl, Lutz Renders, Volker Aßfalg, Stefan Reuter, Daniel Zecher, Jens Werner, Vedat Schwenger, Markus Krautter, Martina Guthoff, Silvio Nadalin, Kai Lopau, Anna Laura Herzog

**Affiliations:** 1 Department of General, Visceral, and Transplantation Surgery, University Medical Center, Mainz, Germany; 2 Department of Internal Medicine, Transplant Nephrology, Faculty of Medicine, University of Münster, Muenster, Germany; 3 Institute of Biostatistics and Clinical Research, University of Münster, Muenster, Germany; 4 Department of Medical Psychology, University Medical Center Hamburg-Eppendorf, Hamburg, Germany; 5 Institute of Medical Informatics, University of Münster, Münster, Germany; 6 Department of Nephrology, University Hospital Heidelberg, Heidelberg, Germany

**Keywords:** living kidney donation, self-reported outcome, surgical complication, surgical techniques, transplantation

## Abstract

**Introduction:**

The German health care system lacks data on surgical complications and self-reported outcomes (SROs) of living donors. The prospective German Living Kidney Donor Registry, SOLKID-GNR aims to improve the assessment of donors’ medical and psychosocial risks.

**Methods:**

Data were collected before (PRE) and 3 months after (POST) living kidney donation from transplantation centers (TCs) and donors via SROs. We reported perioperative complication rates for different surgical techniques and correlated them with donors’ SROs. Datasets of 1020 donors from 30 German TCs were analyzed.

**Results:**

Donor nephrectomy procedures included laparoscopic (57.9%), retroperitoneoscopic (21.4%), open retroperitoneal (16.0%), or open abdominal nephrectomy (4.7%). Perioperative complications reported by TCs ranged from 9.8% (retroperitoneoscopic) to 17.1% (open abdominal), whereas those reported by donors ranged from 12.2% (open retroperitoneal) to 15.0% (open abdominal). Donors were discharged sooner and returned to work earlier after minimally invasive surgery; however, had comparable quality-of-life (QoL) after donation. The physical component summary (PCS) scores of the Short Form–12 (SF-12) were similar between the 4 surgical methods postdonation; however, they were lower in donors with TC- or self-reported complications than in those without. The mental component summary (MCS) scores of the SF-12 were lower in case of self-reported complications only. Despite 12.5% of self-reported complications, 96.4% expressed a willingness to donate again, and 94.1% felt well-informed.

**Conclusion:**

Although the surgical technique does not directly affect donors' QoL shortly after donation, minimally invasive procedures result in shorter hospital stays and a quicker return to work. Self-reported complications have a greater impact on mental QoL than those documented by transplant centers, highlighting the importance of subjective experiences during recovery.

## Graphical abstract

**Figure:**
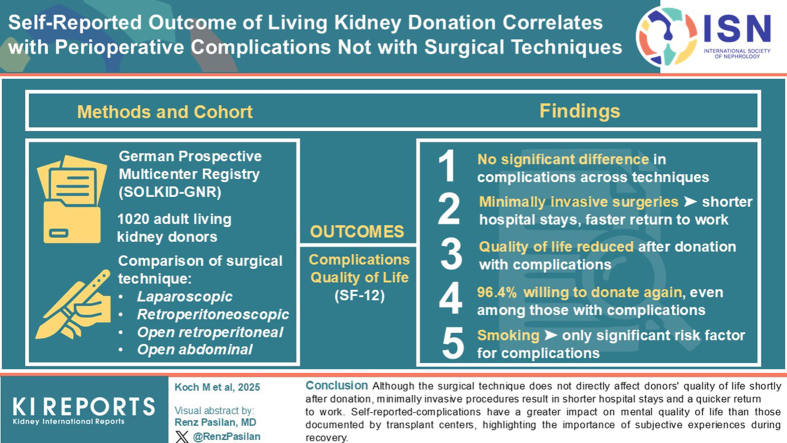

See Commentary on Page 2917

Considering that living donor nephrectomy is a highly elective surgical procedure, the donor’s safety is a major concern. Several studies, registry data, and meta-analyses from around the world suggest that living donor nephrectomy is safe and that the physical burden can be reduced by using minimally invasive surgical techniques.^1^, ^2^, ^3^ The fewest surgical side effects seem to be associated with a retroperitoneoscopic approach.^4^^,^^5^

Although the 2017 Kidney Disease: Improving Global Outcomes Guidelines^6^ declared minimally invasive donor nephrectomy to be standard in several European countries, about 20% of donor nephrectomies in Germany are still performed via an open approach (https://iqtig.org/downloads/auswertung/2019/nls/QSKH_NLS_2019_BUAW_V02_2020-07-14.pdf). In Germany, severe surgical complications with the need for treatment must be reported to the IQTIG (*Institut für Qualität und Transparenz im Gesundheitswesen*). Between 2015 and 2022, reported complication rates were 1.0% to 2.5%.^7^ This rate is quite low compared with data in the literature.^8^^,^^9^

To elucidate surgical complications related to surgical technique and SROs, we analyzed data from SOLKID-GNR. The aim of SOLKID-GNR is to assess the safety of the living kidney donor and provide adequate information for donors and medical professionals in Germany about the physical and mental burden of living kidney donation. In this analysis, we focus on surgical complications reported for the different techniques and on donors’ SROs 3 months after donation. Our main objective was to clarify differences reported by TC and donors’ SRO between different surgical techniques. The secondary objective was to analyze the impact of perioperative complications on donors’ SRO.

## Methods

### Study Design

SOLKID-GNR is a prospective voluntary registry for living kidney donors. It includes not only information provided by the participating TCs, but also donor questionnaires blinded to the treating transplant teams. SOLKID-GNR was registered in the German Clinical Trials Registry (DRKS: 00023532). We received approval from the ethics committee of the Medical Association of Westphalia-Lippe and the University of Münster (2019-732-f-S) and the local ethics committees of the participating TCs before the start of the registry. The registry is funded by the German Federal Ministry of Education and Research (grant no.: 01GY1906) and the Medical Faculty of the University of Münster. Funding sources had no role in study design, data collection, analysis or interpretation.

The design and structure of the registry have been published elsewhere^10^ and are therefore only described briefly. Donor questionnaires were collected up to 4 weeks, medical and demographic data up to 1 year before donation (PRE). For this registry-based study, medical information on surgical techniques, site of nephrectomy, surgical complications, and discharge was used, if it was collected between 4 weeks and 6 months after donation; donor questionnaires had to be completed 8 to 14 weeks after donation (POST).

Between January 2020 and September 2024, 1745 kidney transplantations from living donors were performed in 30 German TCs. Seven TCs did not take part in the registry at this time point. Not all donors could be included in the registry. About 9% of donors had insufficient language proficiency for the donor questionnaires, 11% did not consent to participate, and 7% could not be recruited for other reasons (mostly organizational). Therefore, 1274 donors were included in the registry. For this manuscript only donors were used if medical data from both the PRE and POST time frames were available. These were 1020 out of 1274 donors (Supplementary Figure S1). For 20 of these donors (2%), the donor questionnaires after donation were not available.

### Inclusion Criteria and Informed Consent

To be included, an adult person had to have successfully completed the evaluation process for a living kidney donation and be about to donate. Sufficient knowledge of German, Turkish, or Russian language was necessary to answer donor questionnaires. Donors were informed by the responsible physician and gave their written informed consent to participate in the registry before registry-specific data collection.

### Data Protection, Collection, and Quality

Personal-identifying data were pseudonymized using the pseudonymization service Mainzelliste.^11^^,^^12^ Registry data were collected and managed using REDCap electronic data capture tools^13^^,^^14^ hosted at the medical faculty of the University of Münster. Data collection, pseudonymization, and storage were performed according to the European General Data Protection Regulation (EU-GDPR) guidelines.

Data were collected electronically from TCs as well as directly from donors during out- or in-patient visits before the donation and scheduled outpatient visits to the TC in the context of follow-up after donation.

Data were checked daily for plausibility. Implausible data were compared with the source data during on-site monitoring, or by the TC upon request by the data management via REDCap’s query management tool.

### Donor Questionnaire

Donors were asked to complete standardized questionnaires and answer nonstandardized questions regarding sociodemographic data, SRO, and their experiences of donation. Donors were informed that the questionnaires were not read or analyzed by their transplant team and therefore answers have no impact on their medical treatment. As a standardized questionnaire, we used the SF-12 questionnaire,^15^ which is an abbreviated version of the SF-36 questionnaire, a widely used measure for generic QoL. With the SF-12, we computed 2 summary scale scores, namely the PCS and the MCS. Higher scores indicate better health outcomes. Although there are no universal strict cutoff values for the SF-12, lower scores can reflect reduced health-related QoL. Calculations of the summary scores were done using a validated calculation formula described elsewhere.^15^ The results were compared with age- and gender-specific reference values.^16^ The SOLKID-GNR questionnaires are available on the portal medical-data-models.org as ID 44934 (DOI 10.21961/mdm:44934; validated questionnaires such as the SF-12 are only included as scores).

### Statistical Methods

Categorical variables are presented as absolute and relative frequencies. Continuous variables are presented as mean and SD for normally distributed variables or as median and 25%- and 75%-quartiles in the case of nonnormal distributions.

Comparisons between groups of categorical variables were done using Fisher exact test. A chi-square test was used to compare donors’ smoking status between surgical techniques. Binary variables with a category occurring < 3 times were not tested. Continuous normally distributed variables were compared using the *t* test for 2 groups and analysis of variance was used for > 2 groups.

In continuous nonnormally distributed variables, comparisons were made using the Wilcoxon 2-sample test for 2 groups and the Kruskal-Wallis test for > 2 groups.

A generalized linear mixed model using logit as a link function was used to assess the influence of surgical techniques on the occurrence of surgery-related complications adjusted for variables that were found to be different between surgical techniques (age [> vs. < 65 years], smoking [history of or recent vs. non], and left-sided nephrectomies) and known risk factors (body mass index [> vs. < 30 kg/m^2^), gender, smoking [history of or recent vs. non], and anticoagulation), and the TC as a random variable.

Statistical analysis was done using SAS (version 9.4, SAS Institute in Cary, NC). Missing values were treated as nonexistent. Number of existing values are presented for each variable.

## Results

### Donor Demographics and Surgical Techniques

The 1020 donors in this analysis had a mean age of 54.4 ± 10.2 years. Most were female (*n* = 643, 63.1%), and mean body mass index was 26.1 ± 3.6 kg/m^2^. The mean estimated glomerular filtration rate 2021 (glomerular filtration rate measured according to the CKD-EPI (Chronic Kidney Disease Epidemiology Collaboration) equation of 2021^17^) was 94.6 ± 12.9 ml/min. Of donors, 27.0% (*n* = 262) had hypertension, 45.3% (*n* = 462) had a history of smoking and 12.6% (*n* = 128) stated that they were active smokers. Oral antidiabetic medication and/or anticoagulants such as prophylactic acetylsalicylic acid was used by 0.4% (*n* = 4, all aged > 65 years) and 2.3% (*n* = 22), respectively. Donor characteristics are summarized in Table 1.

**Table 1:** Donor characteristics

Characteristics	All *N* = 1020 (100%)	Open nephrectomy		Minimally invasive nephrectomy	
		OAN *n* = 48 (4.7%)	ORN *n* = 163 (16.0%)	LDN *n* = 591 (57.9%)	RDN *n* = 218 (21.4%)
Donor age (yrs) Mean +/-SD^a^	54.4 ± 10.2	54.0 ± 9.2	57.2 ± 9.4	54.4 ± 10.0	52.5 ± 11.1
Female *n* (%)	643 (63.1)	30 (62.5)	105 (64.4)	372 (63.1)	136 (62.4)
BMI (kg/m^2^) Mean +/− SD	26.1 ± 3.6	25.6 ± 3.5	25.6 ± 3.4	26.1 ± 3.5	26.4 ± 3.8
Right-sided nephrectomies, *n* (%)^a^	464 (45.5)	32 (66.7)	87 (53.4)	229 (38.8)	116 (53.5)
eGFR CKD-EPI 2021 Mean +/− SD (*N* = 974)	94.6 ± 12.9	95.3 ± 12.9	91.7 ± 12.8	95.3 ± 12.9	94.9 ± 12.6
Hypertension, *n* (%) (*N* = 969)	262 (27.0)	13 (29.6)	52 (33.6)	144 (25.7)	53 (25.4)
Diabetes *n* (%) (*N* = 967)	4 (0.4)	0	0	3 (0.5)	1 (0.5)
Any anticoagulation, *n* (%) (*N* = 967)	22 (2.3)	1 (2.3)	4 (2.6)	13 (2.3)	4 (1.9)
Former or active smoking, *n* (%) (*N* = 1019)	590 (57.9)	30 (62.5)	83 (50.9)	342 (58)	135 (61.9)

BMI, body mass index, eGFR CKD-EPI 2021, glomerular filtration rate measured according to the CKD-EPI (Chronic Kidney Disease Epidemiology Collaboration) equation of 2021; LDN, laparoscopic donor nephrectomy; n.s., not significant; OAN, open abdominal nephrectomy; ORN, open retroperitoneal nephrectomy; RDN, retroperitoneoscopic donor nephrectomy.

a Significant difference between groups (*P* < 0.001).

Donor nephrectomy involved the following surgical techniques: laparoscopic or transabdominal donor nephrectomy (LDN) (*n* = 591, 57.9%), retroperitoneoscopic donor nephrectomy (RDN) (*n* = 218, 21.4%), open retroperitoneal nephrectomy (ORN) (*n* = 163, 16.0%) and open abdominal nephrectomy (OAN) (*n* = 48, 4.7%). Donors in the different surgical technique groups were largely comparable, but donors in the ORN group (mean age: 57.2 ± 9.4 years) were slightly older than those in the RDN (mean age: 52.5 ± 11.1 years) and LDN groups (mean age: 54.4 ± 10.0 years) (*P* < 0.001). Accordingly, the estimated glomerular filtration rate was slightly but not significantly lower in the ORN group. The number of right-sided nephrectomies was significantly different between groups, from 38.8% (*n* = 229) in the LDN group to 66.7% (*n* = 32) in the OAN group (*P* < 0.001). For both retroperitoneal techniques (ORN and RDN), right-sided nephrectomies were slightly more common (*n* = 87, 53.4% and *n* = 116, 53.5%, not significant) than in the LDN and OAN groups.

### Outcomes Reported by the TC

The skin-to-skin times were significantly different between groups. The shortest median (Q25–Q75) operation times were in RDN and ORN with 123 (105–155) and 125 (99–175) minutes, respectively, whereas LDN had the longest (185 [153–228] minutes, *P* < 0.001), about 1 hour longer than RDN (*P* < 0.001).

Conversion from a minimally invasive procedure to an open approach occurred in 14 of 582 minimally invasive procedures (2.4%). However, because of low numbers, there was no significant difference between LDN (*n* = 12, 2.8%) and RDN (*n* = 2, 1.3%).

The median (Q25–Q75) hospital stay after RDN was 4 (4–5) days and significantly shorter than after LDN (6 [5–7] days), OAN (7 [6–8] days) and ORN (6 [6–8] days) (*P* < 0.001). Overall, 26 donors (2.8%) were readmitted to the hospital. This rate was not different between groups. Reasons for readmission included surgical complications such as wound healing problems (*n* = 10), ileus or subileus (*n* = 2), pain (*n* = 7), urinary tract infection (*n* = 2), lung embolism (*n* = 1) and other problems (*n* = 4). Data for the different techniques are summarized in Table 2.

**Table 2:** Summary of outcome regarding the different surgical techniques

Outcome parameters	OAN	ORN	LDN	RDN	*P*-value
Outcome reported by transplant centers					
Skin-to-skin time (min) median (Q25–Q75)	134 (92–178)	125 (99–175)	185 (153–228)	123 (105–155)	< 0.001
Reoperations, *n* (%)	0	0	10 (1.8)	3 (1.6)	0.382
Conversion rate, *n* (%)	---	---	12 (2.8)	2 (1.3)	n.a.
Overall complication rate, *n* (%)	6 (17.1)	19 (13.1)	79 (14.3)	19 (9.8)	0.369
Graft injury, *n* (%)	*n* = 3 (4.3)	*n* = 10 (3.5)	*n* = 22 (2.0)	*n* = 11 (2.9)	0.230
Discharge from hospital (d) median (Q25–Q75)	7 (6–8)	6 (6–8)	6 (5–7)	4 (4–5)	< 0.001
Readmission, *n* (%)	1 (2.9)	6 (4.1)	14 (2.5)	5 (2.6)	0.684
Self-reported outcome 8–14 wks after donation (POST)					
Complication rate, *n* (%)	3 (15.0)	12 (12.2)	53 (12.3)	18 (13.0)	0.958
Return to work (wks), mean ± SD	7.0 ± 3.4	7.4 ± 4.2	6.6 ± 3.5	6.5 ± 3.5	0.289
> 50% fit, *n* (%)	14 (70.0)	79 (81.4)	357 (83.8)	118 (84.9)	0.360
No limitations in everyday life, *n* (%)	10 (47.6)	51 (52.0)	230 (53.2)	80 (57.1)	0.767
PCS SF-12, mean ± SD	46.2 ± 10.4	48.5 ± 7.8	48.1 ± 8.4	48.6 ± 8.3	0.636
MCS SF-12, median (Q25–Q75)	57.1 (51.0–60.3)	56.2 (51.6–59.5)	57.5 (53.0;59.4)	57.8 (52.0–59.5)	0.898
Willing to donate again (yes in any case or rather yes), *n* (%)	20 (100)	96 (98.0)	413 (95.8)	135 (96.4)	0.836

LDN, laparoscopic donor nephrectomy; MCS, mental component summary score; n.a., not applicable; n.s., not significant; OAN, open abdominal nephrectomy; ORN, open retroperitoneal nephrectomy; PCS, physical component summary score; RDN, retroperitoneoscopic donor nephrectomy. SF-12, Short Form–12 questionnaire.

No death, dialysis, stroke, or myocardial infarction was reported by the TCs at the POST visit. Overall, 123 of 924 donors (13.3%) had at least 1 complication reported by the TC at the POST assessment. A summary of the complications is presented in Table 3. The most common complications were wound-related complications, affecting 3.1% (*n* = 29) of donors, followed by bleeding or hematoma reported in 2.6% (*n* = 24), with the need for transfusion in 1.4% (*n* = 13). Bleeding with the need for transfusion was higher after OAN (5.7%, *n* = 2) and LDN (2.0%, *n* = 11), whereas no transfusion was reported after ORN or RDN (*P* = 0.011). All other complications were rare (≤ 1%). Complications associated with anesthesia or positioning injury were reported significantly more often in the ORN group. All other complications were similar between the surgical techniques.

**Table 3:** Perioperative complications after donor nephrectomy

Donors with …, *n* (%)	All *N* = 924	OAN *n* = 35	ORN *n* = 145	LDN *n* = 551	RDN *n* = 193	*P*-value
Injury of other organs	4 (0.4)	0	0	4 (0.7)	0	0.549
Any bleeding complication	24 (2.6)	2 (5.7)	1 (0.7)	19 (3.5)	2 (1.0)	0.059
- Bleeding with transfusion	13 (1.4)	2 (5.7)	0	11 (2.0)	0	0.011
Any thrombotic complication	6 (0.7)	1 (2.9)	0	4 (0.7)	1 (0.5)	0.247
- Lung embolism	2 (0.2)	0	0	1 (0.2)	1 (0.5)	n.a.
Any pulmonary complication	8 (0.9)	1 (2.9)	3 (2.1)	3 (0.5)	1 (0.5)	0.109
- Pneumonia	1 (0.1)	0	0	1 (0.2)	0	n.a.
- Pneumothorax	1 (0.1)	0	0	0	1 (0.5)	n.a.
Wound complications	29 (3.1)	0	2 (1.4)	20 (3.6)	7 (3.6)	0.460
- Wound healing problems/ wound infections	22 (2.4)	0	1 (0.7)	14 (2.5)	7 (3.6)	0.315
Other complaints related to the surgical technique	29 (3.1)	1 (2.9)	7 (4.8)	18 (3.3)	3 (1.6)	0.358
- Constipation, ileus, adhesions	9 (1.0)	1 (2.9)	1 (0.7)	6 (1.1)	1 (0.5)	0.481
- Other complications due to anesthesia or positioning	9 (1.0)	0	5 (3.5)	4 (0.7)	0	0.024
Summary I: complications associated with surgical technique	97 (10.5)	5 (14.3)	13 (9.0)	65 (11.8)	14 (7.3)	0.237
complications unlikely related to surgical technique	30 (3.3)	1 (2.9)	6 (4.1)	18 (3.3)	5 (2.6)	0.850
Summary II: All complications	123 (13.3)	6 (17.1)	19 (13.1)	79 (14.3)	19 (9.8)	0.369

LDN, laparoscopic donor nephrectomy; ; n.a., not applicable, no statistical analysis performed if *n* < 3; OAN, open abdominal nephrectomy; ORN, open retroperitoneal nephrectomy; RDN, retroperitoneoscopic donor nephrectomy.

Typical, severe and most common complications are presented as subcategories and number of complications. More than 1 complication per donor is possible.

Overall, 30 donors (3.3%) had complications unlikely to be associated with the surgical technique, such as infections outside the operation field or psychological, cardiac, or neurological complications. Therefore, the 97 donors (10.6%) with complications probably associated with the surgical technique were included in a multivariate analysis on the impact of the surgical technique on complications.

Comparing all 4 techniques, we found no statistically significant difference between the groups (*P* = 0.237) regarding complication rates, which were 7.3% in RDN, 9.0% in ORN, 11.8% in LDN, and 14.3% in OAN. Comparing both transabdominal techniques (LDN and OAN) to both retroperitoneal techniques (RDN and ORN), we found a tendency for fewer complications in the retroperitoneal techniques (8.0%, *n* = 27 vs. 12.0%, *n* = 70) (*P* = 0.059).

Reoperations related to the donation were reported in 13 of 924 donations (1.4%). They were reported solely in minimally invasive techniques (1.8%, *n* = 10 in LDN; and 1.6%, *n* = 3 in RDN), and differences were not significant. Reasons for reoperation were bleeding (*n* = 4), wound infection (*n* = 3), hernia (*n* = 2), intraabdominal abscess (*n* = 1), other (*n* = 1) and unknown (*n* = 2).

We also documented graft injuries with potential impact on the recipient. For example, vascular injuries were reported in 3.8% (*n* = 35) of nephrectomies, and ureter injuries occurred in 1.2% (*n* = 11); no significant differences were found between the surgical techniques.

No significant differences regarding any specific complication were found between right- and left-sided nephrectomies for the donor (12.0%, *n* = 49 vs. 14.2%, *n* = 73) or for the graft (2.8%, *n* = 23 vs. 2.2%, *n* = 23).

In a multivariable regression analysis with the TC as a random effect, we examined whether gender, older age (> 65 years) or body mass index > 30 kg/m^2^ were independent risk factors for perioperative complications associated with the surgical technique; we found no statistical support for this. Furthermore, neither the surgical technique nor the site of nephrectomy were found to affect complication rate (Supplementary Table S1). The only independent significant risk factor we identified for perioperative complications was active or former smoking (*P* = 0.048).

### Donors’ SRO

Concerning outcomes of donation, 12.5% (*n* = 86) of donors reported complications after donation. This aligns with the data reported by the TCs and was not significantly different between surgical techniques (*P* = 0.958). For 670 donors, both SRO and TC medical data were available. Interestingly, for 84 donors with complications reported by the TC, only 39 donors reported having had a complication, whereas the other 45 self-reported as having no complications.

Regarding donors’ well-being, neither the nonstandardized questions nor the SF-12 questionnaire found donor well-being to depend on the surgical techniques. There was no statistically significant difference in response to the nonstandardized question "How fit do you feel for professional or everyday activities?" between the RDN, LDN, ORN, and OAN groups (*P* = 0.360). Only 2.4% (*n* = 16) of donors reported feeling not fit at all, 3.5% (*n* = 24) of donors felt only 25% fit and 10.9% (*n* = 74) felt 50% fit. The answer "totally fit (100%)" was given by 43.9% (*n* = 61) of the donors in the RDN group, 39.2% (*n* = 167) in the LDN group, 34.0% (*n* = 33) in the ORN group, and 35.0% (*n* = 7) in the OAN group. To the question "Are you restricted in your everyday life as a result of the donation?," 57.1% (*n* = 80), 53.2% (*n* = 230), 52.0% (*n* = 51) and 47.6% (*n* = 10) of the donors responded “not at all;” and 8.6% (*n* = 12), 10.0% (*n* = 43), 14.3% (*n* = 14, and 9.5% (*n* = 2) responded "moderately" or "severe" in the RDN, LDN, ORN, and OAN groups, respectively.

**Figure 1:**
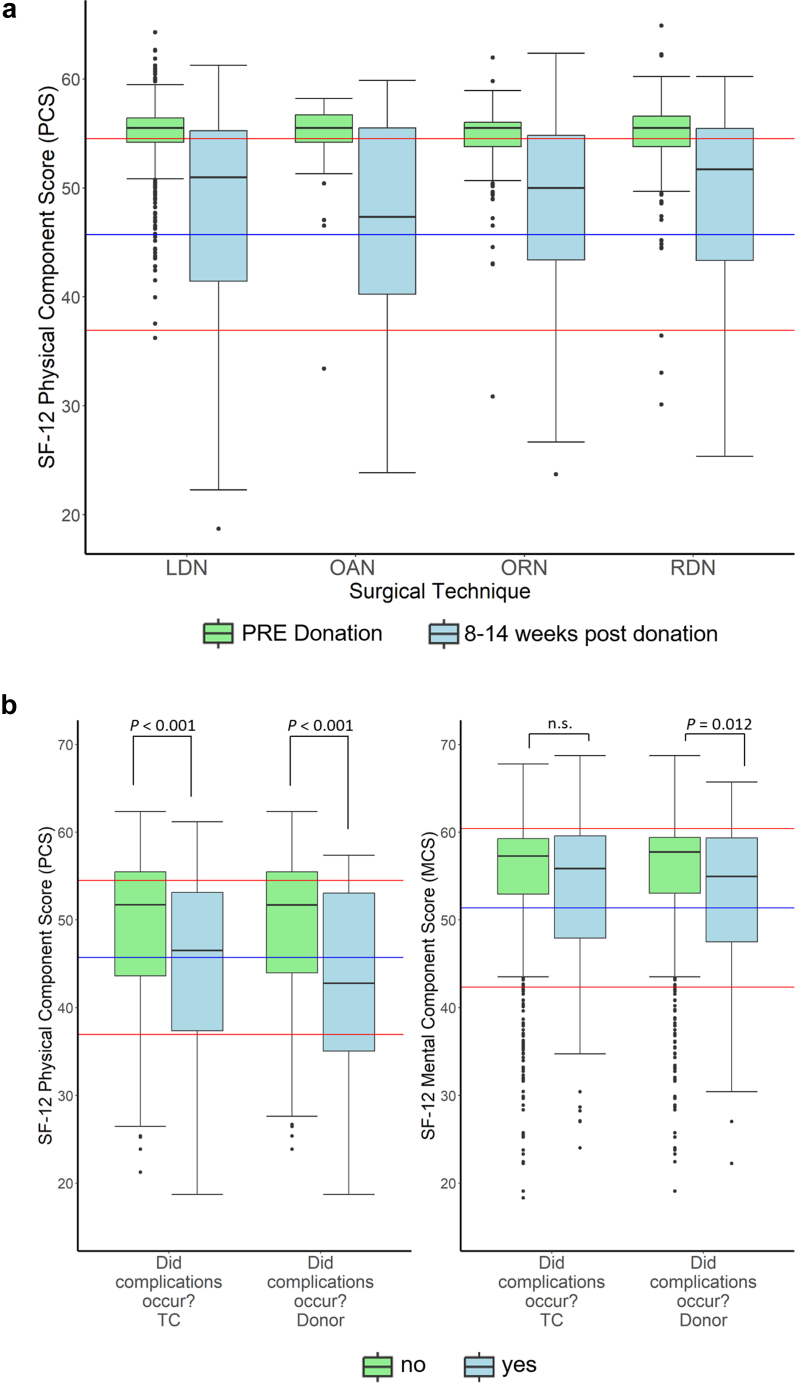
(a) Results for the physical component summary score (PCS) of the SF-12 questionnaire for the different surgical techniques before (PRE) donation (green) versus 8–14 weeks after donation (blue) compared with the mean value (45.72, blue line) ± SD (8.79, red line) for “normal” persons aged 51 to 60 years. Differences between surgical techniques were not significantly different. PRE donation: LDN: *n* = 573; OAN: *n* = 47; ORN: *n* = 162; RDN: *n* = 211. 8–14 weeks POST donation: LDN: *n* = 418; OAN: *n* = 21; ORN: *n* = 98; RDN: *n* = 138. (b) Results for the physical component summary score (PCS, left) and the mental component summary score (MCS, right) of the SF-12 questionnaire for donors without (green) and with (blue) perioperative complications. Included were all donor questionnaires where complications were reported between 8 weeks and 6 months POST donation by the transplant center (TC) or 8–14 weeks for the self-reported complications by the donors. Data were compared with mean values (45.72 [PCS] and 51.38 [MCS], blue line) ± SD (8.79 [PCS] and 9.04 [MCS], red line) for “normal” persons aged 51–60 years. Statistically significant differences were shown within the figure. TC: no: *n* = 713; yes: *n* = 111; Donors: no: *n* = 587; yes: *n* = 84. LDN, laparoscopic donor nephrectomy; n.s., not significant; OAN, open abdominal nephrectomy; ORN, open retroperitoneal nephrectomy; RDN, retroperitoneoscopic donor nephrectomy; SF-12, Short Form–12 questionnaire.

Aligning with donors’ answers to the nonstandardized questions, PCS scores from the SF-12 questionnaire were significantly reduced at POST compared to PRE (mean PRE: 55 ± 3 vs. mean POST: 48 ± 8). The scores were still above or comparable to the mean value of 46 for those aged 51 to 60 years in the reference data^16^ (Figure 1a).

Donors with any perioperative complication, reported either by the donor or the TC, had a significantly lower PCS score than donors without complications (43 ± 10 vs. 49 ± 8, *P* = 0.001; and 44 ± 10 vs. 49 ± 8, *P* = 0.002) (Figure 1b). Donor-reported complications had a stronger effect on mental QoL. The MCS was lower only for donors who reported complications (55 (47–60) vs. 58 (53–59) for those not reporting complications, *P* = 0.012), but not for donors with complications reported by the TC. The results for the different operation techniques are summarized in Table 2.

For the 596 employed donors, the mean time until return to work was between 6.5 and 7.4 weeks. This difference was not statistically significant. Comparing open surgical techniques (OAN and ORN) to minimally invasive techniques (LDN and RDN), we found that donors receiving a minimally invasive surgery returned to work about 1 week (6.6 ± 3.5) earlier than those receiving open surgeries (7.3 ± 4.0) (*P* = 0.093). Donors with ≥ 1 TC-reported complication took longer to return to work (mean: 8.0 ± 3.7 weeks) than donors without complications (6.5 ± 3.5 weeks) (*P* = 0.001). Details are presented in Table 4.

**Table 4:** Employed donors’ return to work

Variables		Number	Sick leave time in wks mean ± SD	*P*-value
All		596	6.7 ± 3.6	
Surgical technique	OAN	21	7.0 ± 3.4	0.289
	ORN	77	7.4 ± 4.2	
	LDN	376	6.6 ± 3.5	
	RDN	122	6.5 ± 3.5	
	Open	98	7.3 ± 4.0	0.064
	Minimally invasive	498	6.6 ± 3.5	
≥ 1 complication	(a) TC-reported (b) Donor-reported	(a) 73 (b) 71	(a) 8.0 ± 3.7 (b) 7.8 ± 3.6	(a) 0.001
No complications	(a) TC-reported (b) Donor-reported	(a) 520 (b) 522	(a) 6.5 ± 3.5 (b) 6.5 ± 3.6	(b) 0.006

LDN, laparoscopic donor nephrectomy; OAN, open abdominal nephrectomy; ORN, open retroperitoneal nephrectomy; RDN, retroperitoneoscopic donor nephrectomy; TC, transplantation center.

In the POST data collection, donors were asked whether they would be willing to donate again; 96.4% (*n* = 664) answered “yes, in any case” or “rather yes,” and only 9 donors answered “no way” or “rather no” (1.3%). This was independent of surgical technique. Reasons given for “no” answers were complications, pain, an overly demanding preoperative evaluation process, insufficient information, postoperative care, and psychological burden.

The large majority of the donors felt well-informed about the donation process. PRE donation, 97.4% (*n* = 993) reported being “very well” or “well” informed about the donation. This did not change after donation: at POST, 94.1% (*n* = 649) answered retrospectively that they had been “very well-” or “well-” informed.

## Discussion

SOLKID-GNR is a prospective scientific registry that includes donors from 30 German TCs performing living kidney donation.^10^ This is the largest prospective analysis on perioperative complications after living donor nephrectomy in Germany and the first to correlate the TC-reported results with outcomes reported by the donors regarding their experience of donation. Notably, donors were included in the registry before donation, making it unlikely that donors with complications were underreported. Only 20 donors did not complete the questionnaires after donation. Furthermore, the donor questionnaires were blinded to the TCs’ attending teams such that donors could report freely on their complications, well-being, and experiences regarding the evaluation and donation process.

In this cohort, the donors’ average age was 54.4 years. This is 10 years older than in most international registry data.^8^^,^^9^ The German population analyzed here was even older than the previous SoLKiD-cohort study, in which participants had a mean age of 52 years.^18^ As such, in our study, more donors had comorbidities such as hypertension (27.0%) and a history of or recent smoking (57.9%). Single centers from Germany and the Netherlands^19^^,^^20^ have also demonstrated this change in risk and age profile of living donors in Europe.

Compared with published data, a higher percentage of living donations in Germany was performed with an open approach.^8^^,^^19^^,^^21^ In this registry, >20% of the donations were performed with an open approach, 21.4% were done retroperitoneoscopically and 57.9% laparoscopically. Including the open retroperitoneal surgeries, over one-third of the living donations were performed through a retroperitoneal approach. Furthermore, more right-sided nephrectomies (45.5%) were performed in Germany compared to other countries^22^; however, some centers seem to hesitate to perform laparoscopic right-sided nephrectomies (38.8%), which might contribute to a higher rate of right open-abdominal nephrectomies (66.7%). It is noteworthy that this side difference was not present in both retroperitoneal techniques.

Despite these particularities in Germany, we demonstrate that living donation is a safe procedure. The overall complication rates reported by the TCs were comparable with published data.^8^^,^^9^^,^^19^^,^^23^^,^^24^ Across all 4 surgical techniques used, 13.3% of donors experienced ≥ 1 complications reported by the TC.

In a large review, researchers showed that retroperitoneoscopic approaches led to lower complication rates than transabdominal laparoscopic approaches,^4^ which does not conflict with our results. For complications likely associated with the surgical technique, we found 11.8% of complications after LDN and 7.3% after RDN. Some single-center studies have reported complication rates as low as 5.6% in very experienced centers^22^^,^^25^; however, this cannot be expected from a registry, including TCs with varying experience.

We did not find that perioperative complications were associated with other risk factors such as obesity (body mass index > 30 kg/m^2^), older age (> 65 years), or gender. Other studies have shown that age alone is not a contraindication for donation,^26^^,^^27^ but data on obesity are controversial. Although a recent study from the Netherlands^19^ did not find higher risks for obese donors, in the US, obesity was associated with a higher complication rate.^8^ We suppose that our donor characteristics may be more comparable with the Netherlands than the US.

Furthermore, we did not detect any disadvantages for donors or recipients of right-sided nephrectomies. This aligns with US registry data^8^ and older data from a large single-center study in Norway, including 76% open surgeries^9^; however, it conflicts with a German cohort study describing right-sided donor nephrectomy as a risk factor for recipient complications.^28^

The only risk factor for donor complications we did confirm was being an active or former smoker. Several types of surgeries have shown that smoking affects perioperative complications^29^; however, it has not consistently been reported as a risk factor in living donors. Although some groups have found no effect, others have reported negative effects on graft survival.^30^, ^31^, ^32^ It needs to be pointed out here that smoking habits were queried by the donors themselves. Therefore, the number of actively smoking donors could be higher than assumed by the TCs.

A unique selling point of our registry is donors’ SROs. Although 12.5% of donors self-reported complications and this value is numerically comparable to the TC-reported complications, the donor’s assessment of complications agreed with that of the TC in less than half of the cases.

The finding that donors do not report the same complications as the TCs is interesting, as is the finding that donor-reported complications more strongly impacted the PCS and especially MCS scores of the SF-12 questionnaire. This is not completely unexpected, because donors might not notice intraoperative complications such as bleeding and the need for transfusions and therefore these might not impact QoL, whereas persistent pain, for example, might not be reported to the TC but may indeed influence QoL.

Although we clearly showed TC-reported differences between the surgical techniques, like significantly longer skin-to-skin times for LDN and earlier hospital discharges for RDN and LDN, donor SROs were not significantly different between the surgical approaches as early as 8 to 14 weeks after donation. This was unexpected, because previous studies have shown than the physical burden of donation can be reduced by minimally invasive techniques when compared with the open approach.^33^, ^34^, ^35^, ^36^ Thus, our donors’ reduced PCS scores were apparently more related to the occurrence of perioperative complications than to the surgical technique.

One cannot expect that donors feel completely unaffected by the donation 8 to 14 weeks after donation. One study showed that donors’ PCS scores from the SF-36 were lower 2 months after donation, whereas their MCS scores were unaffected.^18^ This corresponds with our data; we also demonstrated that donors' self-reported complications correlate with a reduced MCS.

To classify the PCS scores in our cohort, we can compare the results with a small cohort of patients after flank incision due to urological indication. In that cohort, the PCS score of patients without chronic pain was 50, whereas in patients with chronic pain it was 40.1.^37^ Our donors’ PCS scores fall in the upper range of these results; however, notably, the PCS scores from donors in the minimally invasive surgery groups LDN and RDN were not better. Previously, researchers showed that after laparoscopic tumor nephrectomy, patients needed 3 months to recover from surgery.^38^ Fortunately, most donors’ PCS scores seem to recover after 1 year.^18^^,^^21^ As such, we will follow up on our cohort.

Notably though, an important finding from our analysis is that despite a relevant complication rate after living donor nephrectomy and a negative physical impact for several weeks (only 39.3% of donors felt totally fit at POST), nearly all donors (96.4%) expressed a willingness to donate again.

Before donation, 97.4% of donors stated that they felt well- or very well-informed about the donation, which was confirmed at POST by 94.1% of donors responding the same way. We think this is a very positive result, because it shows excellent work by the TCs in informing donors, and it highlights donors’ capability to understand and accept the risks of living kidney donation.

Limitations of our data analysis are that the registry is voluntary and restricted to donors who speak German, Turkish, or Russian. Not all, but the vast majority of German TCs participated in the registry and prospectively included 79% of donors fulfilling the inclusion criteria. Because of the prospective character of the registry, donors were enrolled before donation, where the outcome was not foreseeable, which eliminated a possible positive selection bias of donors who had a living donation without complications. We cannot rule out that some donors withdraw consent after donation; however, the numbers reported are very small. Due to the monitoring of medical data, it is unlikely that data from the TC includes mistakes to a larger extent; however, the donor questionnaires could not be checked for misunderstandings or intentional mistakes. We assume that most donors answered honestly.

### Interpretation

In conclusion, data from the largest German multicenter registry indicates that all surgical techniques used for living kidney donation in Germany are safe and do not significantly differ in perioperative complication rates or donors’ self-reported QoL early after donation. About 3 months after donation, most donors expressed a willingness to donate again. Finally, based on our results, we presume that donors do not have the same perception of complications as treating physicians, because donors’ physical and mental well-being correlated more with self-reported than TC-reported complications.

## Appendix

### List of the members of the SOLKID-GNR Registry Group (in alphabetical order of the transplantation center)

Anja Mühlfeld, MD, Division of Nephrology and Immunology, University Hospital RWTH Aachen, Aachen, Germany; Florian Sommer, MD, Klinik für Allgemein-, Viszeral- und Transplantationschirurgie – Transplantationszentrum, Universitätsklinikum Augsburg, Augsburg, Germany; Aydin Er, MD, Medizinische Klinik – Transplantationszentrum, Universitätsklinikum Augsburg, Augsburg, Germany; Klemens Budde, MD and Lutz Liefeld, MD, Department Nephrology and Medical Intensive Care, Charité University Berlin/Campus Mitte, Berlin, Germany; Fabian Halleck, MD and Mira Choi, MD, Department Nephrology and Medical Intensive Care, Charité University Berlin/Campus Virchow, Berlin, Germany; Julian Stumpf, MD, and Juliane Putz, MD, Bereich Nephrologie, Medizinische Klinik und Poliklinik III, University Hospital Carl Gustav Carus, TU Dresden, Dresden, Germany; Johannes Stegbauer, MD and Susanne Mende, MD, Department of Nephrology, Faculty of Medicine, University Hospital, Heinrich-Heine-University, Duesseldorf, Germany; Mario Schiffer, MD, Medizinische Klinik 4, Nephrologie und Hypertensiologie, Universitätsklinikum Erlangen, Erlangen, Germany; Katharina Heller, MD, Medizinische Klinik 4, Nephrologie und Hypertensiologie, Transplantationszentrum, Universitätsklinikum Erlangen, Erlangen, Germany; Andreas Kribben, MD, Klinik für Nephrologie, Universitätsklinikum Essen, Universität Duisburg-Essen, Essen, Germany; Bernd Jänigen, MD, Department of General and Digestive Surgery, Section of Transplant Surgery, Faculty of Medicine, University of Freiburg, Freiburg, Germany; Johanna Schneider, MD, Department of Medicine IV, University Freiburg Medical Center, Faculty of Medicine, University of Freiburg, Freiburg, Germany; Rolf Weimer, MD and Hristos Karakizlis, MD, Department of Internal Medicine, Nephrology and Renal Transplantation, University Clinic of Giessen and Marburg (UKGM), Campus Giessen, Giessen, Germany; Ulrich Pein, MD, University Hospital Halle (Saale), Department of Internal Medicine, Halle (Saale), Germany; Lutz Fischer, MD, Klinik und Poliklinik für Viszerale Transplantationschirurgie und Universitäres Transplantations Centrum, Uniklinikum Hamburg-Eppendorf, Hamburg, Germany; Malte Kluger, MD, III. Med. Klinik und Universitäres Transplantations Centrum, Uniklinikum Hamburg-Eppendorf, Hamburg, Germany; Peter Weithofer, MD and Volker Kliem, MD, Nephrologisches Zentrum Niedersachsen, Klinikum Hann. Münden GmbH, Hann. Münden, Germany; Martin Zeier, MD, Department of Nephrology, University Hospital Heidelberg, Heidelberg, Germany; Mandy Schlosser, MD and Gunter Wolf, MD, Klinik für Innere Medizin III, Universitätsklinikum Jena, Jena, Germany; Thomas Rath, MD, Abteilung für Nephrologie und Transplantationsmedizin, Westpfalz-Klinikum Kaiserslautern, Kaiserslautern, Germany; Christian Mönch, MD, Klinik für Allgemein-, Viszeral-, Kinder- und Transplantationschirurgie, Westpfalz-Klinikum Kaiserslautern, Kaiserslautern, Germany; Kevin Schulte, MD and Friedrich A. von Samson-Himmelstjerna, MD, Department of Nephrology and Hypertension, University Hospital Schleswig-Holstein, Kiel, Germany; Dirk Stippel, MD, Department of General, Visceral, Cancer and Transplant Surgery, University of Cologne Faculty of Medicine and University Hospital Cologne, Cologne, Germany; Christine Kurschat, MD, Department II of Internal Medicine and Center for Molecular Medicine Cologne, University of Cologne, Faculty of Medicine and University Hospital Cologne, Cologne, Germany; Ana Harth, MD and Christian Jungck, MD, Medizinische Klinik I (Klinik für Nephrologie, Transplantationsmedizin und internistische Intensivmedizin), Kliniken der Stadt Köln, Cologne, Germany; Anette Bachmann, MD and Antje Weimann, MD, Department für Innere Medizin, Neurologie und Dermatologie, Klinik für Endokrinologie/Nephrologie, Universitätsklinikum Leipzig, Leipzig, Germany; Martin Nitschke, MD and Figen Cakiroglu, MD, Medizinische Klinik I, Nephrologie Universitätsklinikum Schleswig-Holstein, Campus Lübeck, Lübeck, Germany; Julia Weinmann-Menke, MD, I. Medizinische Klinik und Poliklinik, Universitätsmedizin Mainz, Mainz, Germany; Birgit Kortus-Götze, MD and Joachim Hoyer, MD, Klinik für Innere Medizin, Nephrologie, Universitätsklinikum Gießen und Marburg, Standort Marburg, Marburg, Germany; Stephan Kemmner, MD, Transplantationszentrum, Klinikum der Universität München, Munich, Germany; Manfred Stangl, MD, Klinik für Allgemeine, Viszeral-, Transplantations-, Gefäß- und Thoraxchirurgie, Klinikum der Universität München, Campus Großhadern, Munich, Germany; Lutz Renders, MD, Abteilung für Nephrologie, TUM Universitätsklinikum, Klinikum rechts der Isar, Munich, Germany; Volker Aßfalg, MD, Klinik und Poliklinik für Chirurgie, TUM Universitätsklinikum, Klinikum rechts der Isar, Munich, Germany; Stefan Reuter, MD, Department of Internal Medicine, Transplant Nephrology, University Hospital Münster, Muenster, Germany; Daniel Zecher, MD, Department of Nephrology, University Hospital Regensburg, Regensburg, Germany; Jens Werner, MD, Department of Surgery, University Hospital Regensburg, Regensburg, Germany; Vedat Schwenger, MD and Markus Krautter, MD, Klinik für Nieren-, Hochdruck- und Autoimmunerkrankungen, Klinikum der Landeshauptstadt Stuttgart gKAöR, Katharinenhospital, Stuttgart, Germany; Martina Guthoff MD, Department of Diabetology, Endocrinology, Nephrology, Section of Nephrology and Hypertension, University of Tübingen, Tübingen, Germany; Silvio Nadalin, MD, Department of General Visceral and Transplant Surgery, University of Tübingen, Tübingen, Germany; Kai Lopau, Department of Nephrology, University Hospital Würzburg, Würzburg, Germany; and Anna Laura Herzog, MD, Transplantation center, University Hospital Würzburg, Würzburg, Germany.

## Disclosure

All the authors declared no competing interests.

## Supplementary Material

Supplementary File (PDF) **Figure S1.** Flow chart of number of individuals and reasons why donors did not participate or datasets got excluded for this study. **Table S1.** Multivariate regression analysis for perioperative complications. STROBE Checklist.

## Footnotes

Supplementary File (PDF)
**Figure S1.** Flow chart of number of individuals and reasons why donors did not participate or datasets got excluded for this study. **Table S1.** Generalized linear mixed model using logit as a link function and the surgery centre as a random variable. STROBE Checklist.
