# Supplementary material for: Self-Reported Outcome of Living Kidney Donation Correlates With Perioperative Complications not With Surgical Techniques
Source: Kidney Int Rep. 2025 Jul 2;10(9):3058–69. doi: 10.1016/j.ekir.2025.06.052 (PMC12446988; doi:10.1016/j.ekir.2025.06.052)
Supplement: Supplementary File (PDF) — Figure S1. Flow chart of number of individuals and reasons why donors did not participate or datasets got excluded for this study. Table S1. Multivariate regression analysis for perioperative complications. STROBE Checklist. [file mmc1.pdf]

Supplementary Figure S1: Flow chart of number of individuals and reasons why donors did not participate or data sets got excluded for this study. Transplantation centre (TC); Living kidney donor (LKD);

Footnote to Figure S1: [a] Especially the standardized psycho-social questionnaires are hard to understand even for fluently German speaking donors, if German was not mothers tong. Therefore, 9% of the donors could not be included in the registry. [b] Participation in the registry was absolutely voluntary. 11% of the donors did not consent in participating in the registry, including filling out several questionnaires on a tablet. [c] 7% of the donors were not included by the centers, e.g. due to staff shortage or unavailability of tablet computer. The participation in the registry was completely voluntary for the centers and the donors.

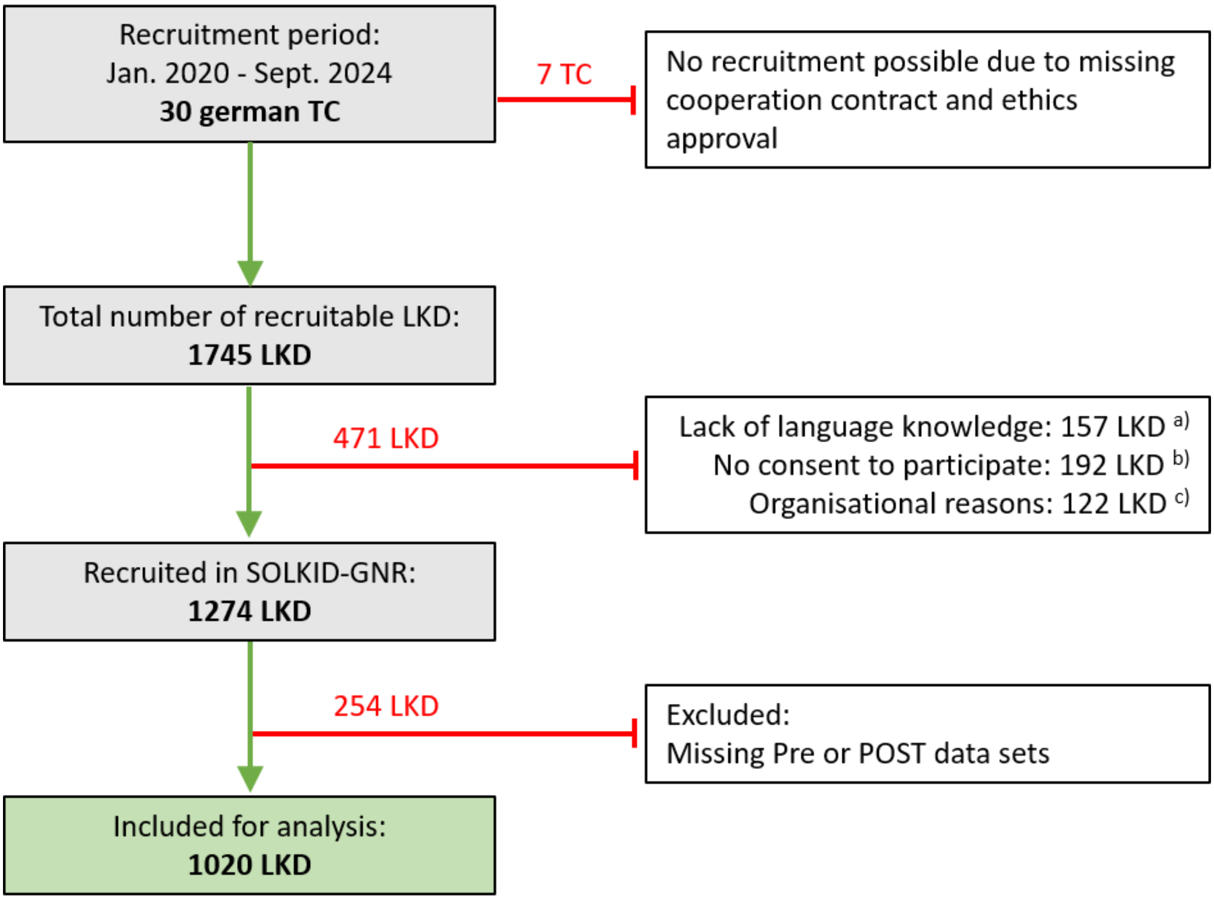

Supplementary Table S1: Generalized linear mixed model using logit as a link function and the surgery centre as a random variable

Effect	Comparison	Odds Ratio	Lower Confidence Limit for Odds Ratio	Upper Confidence Limit for Odds Ratio	P-Value
Gender	Male vs. female	1.061	0.682	1.650	0.793
Medication anticoagulants	Yes vs. No	2.510	0.846	7.449	0.097
surgical technique	LDN vs. RDN	1.677	0.861	3.267	0.368
surgical technique	ORN vs. RDN	1.286	0.537	3.081	0.368
surgical technique	OAN vs. RDN	2.210	0.691	7.067	0.368
Site of nephrectomy	Left vs. Right	1.452	0.922	2.287	0.107
BMI	BMI ≥ 30kg/m^2 vs. BMI < 30kg/m^2	0.973	0.516	1.838	0.934
Age	Age ≥ 65 Years vs. Age < 65 Years	1.069	0.581	1.965	0.830
Smoking status	Recent/history of Smoking vs. No smoking	1.592	1.004	2.522	0.048

Open abdominal nephrectomy (OAN); open retroperitoneal nephrectomy (ORN); retroperitoneoscopic donor nephrectomy (RDN); laparoscopic donor nephrectomy (LDN).

STROBE Statement—checklist of items that should be included in reports of observational studies

	Item No.	Recommendation	Page No.	Relevant text from manuscript
**Title and abstract**	1	(*a*) Indicate the study's design with a commonly used term in the title or the abstract	5	prospective German Living Kidney Donor Registry
		(*b*) Provide in the abstract an informative and balanced summary of what was done and what was found	5	done
**Introduction**				
Background/rationale	2	Explain the scientific background and rationale for the investigation being reported	7	done
Objectives	3	State specific objectives, including any prespecified hypotheses	7	This is clarified in the last paragraph of the introduction
**Methods**				
Study design	4	Present key elements of study design early in the paper	6-9	Provided in the Method section
Setting	5	Describe the setting, locations, and relevant dates, including periods of recruitment, exposure, follow-up, and data collection	6-9	Provided in the Method section
Participants	6	(*a*) *Cohort study*—Give the eligibility criteria, and the sources and methods of selection of participants. Describe methods of follow-up<br>*Case-control study*—Give the eligibility criteria, and the sources and methods of case ascertainment and control selection. Give the rationale for the choice of cases and controls<br>*Cross-sectional study*—Give the eligibility criteria, and the sources and methods of selection of participants	6-7	Described in the Method section \`Study design\`and Ìnclusion criteria and informed consent
		(*b*) *Cohort study*—For matched studies, give matching criteria and number of exposed and unexposed<br>*Case-control study*—For matched studies, give matching criteria and the number of controls per case		not relevant

Variables	7	Clearly define all outcomes, exposures, predictors, potential confounders, and effect modifiers. Give diagnostic criteria, if applicable	8-9	Provided in the Method section
Data sources/ measurement	8*	For each variable of interest, give sources of data and details of methods of assessment (measurement). Describe comparability of assessment methods if there is more than one group	*8-9*	*Donor questionnaires*
Bias	9	Describe any efforts to address potential sources of bias	15	Last paragraph of the Discussion section
Study size	10	Explain how the study size was arrived at	6-7	Method section `Study design` and Supplementary Fig.1

Continued on next page

Quantitative variables	11	Explain how quantitative variables were handled in the analyses. If applicable, describe which groupings were chosen and why	8-9	Described in Method section `Statistical methods´
Statistical methods	12	(*a*) Describe all statistical methods, including those used to control for confounding	8-9	Described in Method section `Statistical methods
		(*b*) Describe any methods used to examine subgroups and interactions	8-9	Described in Method section `Statistical methods
		(*c*) Explain how missing data were addressed	8-9	Described in Method section `Statistical methods
		(*d*) *Cohort study*—If applicable, explain how loss to follow-up was addressed<br>*Case-control study*—If applicable, explain how matching of cases and controls was addressed<br>*Cross-sectional study*—If applicable, describe analytical methods taking account of sampling strategy		Not applicable
		(*e*) Describe any sensitivity analyses		-
**Results**				
Participants	13*	(a) Report numbers of individuals at each stage of study—eg numbers potentially eligible, examined for eligibility, confirmed eligible, included in the study, completing follow-up, and analysed	6-7	Included in Method section `Study design
		(b) Give reasons for non-participation at each stage	7	Included in Method section `Study design + Supplementary Fig.1
		(c) Consider use of a flow diagram	7	Supplementary Fig. 1
Descriptive data	14*	(a) Give characteristics of study participants (eg demographic, clinical, social) and information on exposures and potential confounders	9 + 19	Done in the Donor demographics section and Table 1
		(b) Indicate number of participants with missing data for each variable of interest	8-9 + 19-21	Number of donors included in the analysis was specified for each variable; Table1-4
		(c) *Cohort study*—Summarise follow-up time (eg, average and total amount)		
Outcome data	15*	*Cohort study*—Report numbers of outcome events or summary measures over time	*19-21*	Numbers are given in Tab 2, 3, 4

		Case-control study—Report numbers in each exposure category, or summary measures of exposure		-
		Cross-sectional study—Report numbers of outcome events or summary measures		-
Main results	16	(*a*) Give unadjusted estimates and, if applicable, confounder-adjusted estimates and their precision (eg, 95% confidence interval). Make clear which confounders were adjusted for and why they were included	9-12	Results section
		(*b*) Report category boundaries when continuous variables were categorized		-
		(*c*) If relevant, consider translating estimates of relative risk into absolute risk for a meaningful time period		-

Continued on next page

Other analyses	17	Report other analyses done—eg analyses of subgroups and interactions, and sensitivity analyses	8-9	Described in Method section `Statistical methods´ and Suppl. Table 1
**Discussion**				
Key results	18	Summarise key results with reference to study objectives	12-15	Discussion section
Limitations	19	Discuss limitations of the study, taking into account sources of potential bias or imprecision. Discuss both direction and magnitude of any potential bias	15	Last paragraph in Discussion section
Interpretation	20	Give a cautious overall interpretation of results considering objectives, limitations, multiplicity of analyses, results from similar studies, and other relevant evidence	15	Interpretation section
Generalisability	21	Discuss the generalisability (external validity) of the study results	15	done
**Other information**				
Funding	22	Give the source of funding and the role of the funders for the present study and, if applicable, for the original study on which the present article is based	15	done

*Give information separately for cases and controls in case-control studies and, if applicable, for exposed and unexposed groups in cohort and cross-sectional studies.

Note: An Explanation and Elaboration article discusses each checklist item and gives methodological background and published examples of transparent reporting. The STROBE checklist is best used in conjunction with this article (freely available on the Web sites of PLoS Medicine at http://www.plosmedicine.org/, Annals of Internal Medicine at http://www.annals.org/, and Epidemiology at http://www.epidem.com/). Information on the STROBE Initiative is available at www.strobe-statement.org.
